# Optimized Setup and Protocol for Magnetic Domain Imaging with *In Situ* Hysteresis Measurement

**DOI:** 10.3791/56376

**Published:** 2017-11-07

**Authors:** Jun Liu, John Wilson, Claire Davis, Anthony Peyton

**Affiliations:** ^1^Advanced Steel Research Centre, Warwick Manufacturing Group, University of Warwick; ^2^School of Electrical and Electronic Engineering, University of Manchester

**Keywords:** Bioengineering, Issue 129, Magnetic domain, Bitter method, Steel, *BH* loop, Dynamic domain imaging, *in situ*, Domain wall movement

## Abstract

This paper elaborates the sample preparation protocols required to obtain optimal domain patterns using the Bitter method, focusing on the extra steps compared to standard metallographic sample preparation procedures. The paper proposes a novel bespoke rig for dynamic domain imaging with *in situ BH* (magnetic hysteresis) measurements and elaborates the protocols for the sensor preparation and the use of the rig to ensure accurate *BH* measurement. The protocols for static and ordinary dynamic domain imaging (without *in situ BH* measurements) are also presented. The reported method takes advantage of the convenience and high sensitivity of the traditional Bitter method and enables *in situ BH* measurement without interrupting or interfering with the domain wall movement processes. This facilitates establishing a direct and quantitative link between the domain wall movement processes–microstructural feature interactions in ferritic steels with their *BH* loops. This method is anticipated to become a useful tool for the fundamental study of microstructure–magnetic property relationships in steels and to help interpret the electromagnetic sensor signals for non-destructive evaluation of steel microstructures.

**Figure:**
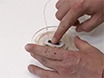


## Introduction

A variety of electromagnetic (EM) sensors have been developed or commercialized for evaluating and monitoring microstructure, mechanical properties or creep damage in ferritic steels during industrial processing, heat treatment or service exposure^1^,^2^. These sensors operate in a non-destructive and non-contact fashion and are based on the principle that microstructural changes in ferritic steels alter their electrical and magnetic properties. In order to interpret the EM signals in terms of microstructures, one has to link the EM signals to their causal magnetic properties and then to the microstructure of the materials. Relationships between the various EM sensor signals such as mutual inductance for multi-frequency EM sensors and the EM properties (*e.g.* relative permeability and conductivity) are well established in electromagnetics research with analytical relationships having been reported for several typical sensor geometries^3^. However, the relationships between the EM or magnetic properties (*e.g.* the initial permeability, coercivity) and specific microstructures still remain more or less empirical, qualitative or, in many cases, unavailable, particularly when there are more than one type of microstructural features of interest affecting the magnetic behavior^4^.

Ferromagnetic materials contain magnetic domains, consisting of aligned magnetic moments, separated by domain walls (DWs). As a magnetic field is applied, domains will be re-aligned through DW motion, domain nucleation and growth, and/or domain rotation. More details on domain theory can be found elsewhere^5^. Microstructural features such as precipitates or grain boundaries can interact with these processes and hence affect the magnetic properties of ferromagnetic materials^4^,^6^,^7^,^8^. The different microstructural features in steels and their magnetic properties can affect the domain structures and the DW movement process when a magnetic field is applied. It is necessary to look into the magnetic domain structure and the interaction between DWs and microstructure features under different applied fields and frequencies in order to establish a fundamental link between the microstructure and magnetic properties in steels.

Magnetic hysteresis loops or *BH* loops can describe the fundamental magnetic properties of the materials such as the coercivity, remanence, differential and incremental permeability, amongst others. *BH* loop analysis has become a useful non-destructive testing (NDT) technique for evaluation of microstructure and mechanical properties of ferritic steels^9^,^10^. The *BH* loop is a plot of the magnetic flux density in the material under inspection (*B*) versus the applied magnetic field (*H*). As a magnetic field is induced in the sample by an excitation coil provided with a time varying current, *B* is measured using a second coil encircling the sample under inspection, while *H* is measured using a magnetic field sensor (commonly a Hall sensor) placed close to the surface of the sample. The most accurate measurement of a material's *BH* characteristics can be made using a closed magnetic circuit, like that presented by a ring sample, but other methods such as the use of a separate excitation core can yield satisfactory results. It is of both great scientific significance and practical value to be able to carry out *in situ* observation of the DW movement processes during magnetic measurements and to directly link these to the magnetic properties and microstructure. Meanwhile, it is very challenging to do either the domain observation or the magnetic measurements without affecting the other.

Amongst various domain imaging techniques, the Bitter method, i.e. using fine magnetic particles to reveal magnetic DWs, has some obvious advantages including easy set-up and high sensitivity^11^. Due to the use of a medium, *e.g. *ferro-fluid, it takes a lot of experience and time to obtain high quality patterns and consistent results using Bitter methods. Standard metallographic sample preparation, intended and optimized for optical microscopy (OM) and scanning electron microscopy (SEM), usually yields unsatisfactory Bitter patterns for many steels because the Bitter method is less tolerant to the residual subsurface damage and the associated artificial effects than OM and SEM. There are possible artificial effects due to poor application of ferro-fluid. This paper details additional sample preparation procedures, compared to the standard metallographic ones, preparation and application of ferro-fluid, observation of domain structures using optical microscopes and the method for *in situ* magnetic measurement.

Many studies on the observation of domain structures in single crystals (*e.g.* Si-iron^12^) or grain-oriented Si electrical steels have been reported^13^. In these materials only a small number of microstructural features (i.e. grain/crystal orientation and grain boundaries) were involved and the domain structures are relatively coarse (with the domain width being on the order of 0.1 mm^12^). In this paper, domain patterns in polycrystalline ferritic steels, including a plain low carbon steel (0.17 wt% C) have been observed and reported. The low carbon steel has much finer grain size (approximately 25 µm on average in equivalent circular diameter) and finer domain structure (with the domain width on the order of micrometers) than the electrical steels and hence show complex interactions between the various microstructural features and DW movement processes.

This paper proposes a novel bespoke rig for dynamic domain imaging using the Bitter method with *in situ BH* (magnetic hysteresis) measurements. The reported method takes advantage of the convenience and high sensitivity of the traditional Bitter method and enables *in situ *BH measurement without interrupting or interfering with the domain wall movement processes. This facilitates establishing a direct and quantitative link between the domain wall movement processes-microstructural feature interactions in ferritic steels with their *BH* loops. This method is anticipated to become a useful tool for the fundamental study of microstructure-magnetic property relationships in steels and to help interpretation of electromagnetic sensor signals for non-destructive evaluation of steel microstructures.

## Protocol

### 1. Preparation of Specimens for Domain Imaging with *In Situ* *BH* Measurement

1. Machine two U-shaped parts (Parts A and B) from the steel of interest, as shown in Figure 1, by Electrical Discharge Machining (EDM). Note the difference between the two parts,* i.e.* 1 mm thicker horizontal part and the 1 mm chamfering in Part A, is designed to ensure a known and needed thickness (1.5 mm in this paper) after the sample (Part A) is mounted and ground (see Figure 1 for the dimensions and procedures 2.1 - 2.4 for more details).

### 2. Preparation of Metallographic Samples

1. **Hot-compression mount Part A, preferably using the compounds that produce a transparent mount.** CAUTION: Use the correct amount of compounds to avoid damaging the sample during compression mounting. The final thickness of the mount should be 5 - 10 mm greater than the height of the sample. It is worth noting there might be residual stress caused by the compression mounting, which might then lead to some effects on the domain structure.
  1. Place Part A, with the two legs facing upwards, into the mold of the compression-mounting machine.
  2. Add about 20 mL of methyl methacrylate compound powder into the mold.
  3. Start a mounting cycle with the following parameters: heating time - 4.5 min, cooling time - 4 min, pressure - 290 Bar and temperature - 180 °C.
  4. Take out the mount when the cycle is finished and check the thickness. It should be between 20 - 25 mm.
2. Grind the side of the mounted sample with the two legs of the U-shaped sample facing it using 320 grit SiC paper on a grinding machine until the base of the legs are revealed on the surface. NOTE: Automated grinding is recommended to ensure the two flat surfaces of the mount are parallel after grinding.
3. Grind the other side of the mount and check frequently until the flat part of the U-shaped sample surface shows, grind until the rectangular surface is revealed.
4. Measure the length of the revealed sample using a caliper and continue grinding carefully and measure frequently. The revealed sample length will initially increase with grinding (typically slightly over 23 mm when the initial rectangular shape is revealed). Stop grinding as soon as the length reaches 25.05 ± 0.05 mm. At this point, the polished sample will have the same dimensions as the sensor part (Part B in Figure 1) i.e. 25 mm length and 1.5 mm thickness. This procedure, together with the designed chamfering of the sample (Part A Figure 1), gives the known and needed sample thickness, within a tolerance of about tens of microns, after grinding.
5. Polish the sample according to the standard metallographic sample preparation procedures for soft steels 14. CAUTION: Do not re-grind the sample, as this will change the sample thickness and therefore cause inaccurate *BH* measurement.
6. Etch the polished sample using a cotton swab with suitable reagent (e.g. 2% nital for pure iron or low carbon steel) for 1 - 5 s until the polished surface turns matte.
7. Check the sample under an optical microscope. An effective etching will reveal the microstructure of the sample clearly.
8. Polish the sample again using 1 µm diamond polishing agent until the etched surface layer is completely removed. Check under the microscope if not sure.
9. Repeat step 2.6 - 2.8 for 4 - 6 times. This removes any work hardened surface layer.
10. Finish the polishing using alumina suspension for 2 min. NOTE: The experiment can be paused here.

### 3. Preparation of the Flux Density (B) Measurement Coil

1. **Make the sensor using Part B, shown in Figure 1.**
  1. Wrap a layer of double sided tape along base of the U shape (*i.e.* longest side) of Part B.
  2. Using 0.20 mm diameter enameled copper wire, wrap a single layer, 50 turn coil around the longest side of part B, leaving around 100 mm of wire at each end of the coil.
  3. Remove the enamel from the last 20 mm of each end of the wire using 800 grit sandpaper.
2. **Check for electrical short circuits between coil and sample.**
  1. Take a multimeter and set it to test for continuity. Touch one probe to Part B and the other to the end of one wire. NOTE: There should be no continuity between coil and sample, if there is continuity between coil and sample, the wire has shorted to the sample and the coil should be removed and re-applied.

### 4. Set Up the Domain Imaging Rig

1. **Install/fix the samples on the test rig shown in Figure 2.**
  1. Place the front plate shown in Figure 2(a) on a flat surface and fit the mounted sample into the hole in the front plate.
  2. Apply hot melt from a glue gun around the circumference of the mounted sample to hold it in place.
  3. Insert Part B through the excitation coils into the bottom of the sample holder; the sample should protrude from the top of the sample holder by around 1 mm.
  4. Fix the back plate onto the back of the sample holder and loosely tighten the bottom nuts, ensuring that the Hall sensor is aligned with the sample by visual inspection.
  5. Apply exciting current to the excitation coil, which forms an electromagnet, for easy assembly and alignment.
  6. Align the top of Part A with the bottom of Part B, as in Figure 2, with the help of the magnetic force of the aforementioned electromagnet (maximum force felt at perfect alignment) as well as by visual inspection if the sample mount is transparent. Accurate coupling of the Part A and Part B is important to the accuracy of the BH loop measurement. See Discussion for a more detailed explanation.
  7. Bolt the top plate to the sample holder.
  8. Tighten the bottom nuts to apply pressure between Part A and Part B. It is worth noting that overtightening may cause stress within the materials and hence stress-induced effects on the domain structure. The test rig should now look like Figure 2b.
2. **Level the sample for a consistently good focusing across the field of vision. This step is highly recommended if an objective lens of 50 times or higher is used and must be done before applying the ferro-fluid.**
  1. Put a piece of modeling clay the size of a cherry onto a clean glass slide.
  2. Place the test rig on top of the modeling clay with the sample approximately center aligning with the rig.
  3. Put three sheets of lens tissue on top of the sample surface for protection.
  4. Level the whole test rig using a levelling press for microscopy with the sample approximately center aligned with the press.

### 5. Magnetic Domain Imaging

1. **Dilution of oil-based ferro-fluid.**
  1. Draw 1 mL of oil-based ferro-fluid using a pipette and add it to a 5 mL vial.
  2. Add 0.5 mL of the original solvent (hydrocarbon oil) for the ferro-fluid into the vial.
  3. Shake for 10 s.
2. **Application of ferro-fluid on the sample.**
  1. Draw a single drop (about 0.25 mL) of the ferro-fluid using a pipette and apply on the sample surface.
  2. Put a clean microscope slide on the sample and slowly slide the glass slide off the sample surface to form a thin and uniform layer. A good finish should be semi-transparent with an amber color.
3. **Static domain imaging**
  1. Observe the domain pattern under a light microscope before the ferro-fluid dries out. Use ample lighting and a small aperture (by adjusting the aperture diaphragm) for optimal contrast. CAUTION: Avoid exposure of the ferro-fluid to strong light longer than necessary as this may dry the ferro-fluid.
  2. Wipe or rinse with acetone to remove the ferro-fluid after domain imaging.
  3. Clean the sample surface thoroughly and dry the sample after experiments.
4. **Dynamic domain imaging**
  1. Attach a high-speed video camera to the microscope.
  2. Apply a magnetic field to the sample to make the DWs move. The present test rig can be used to apply a field of up to 4 kA/m in parallel with the sample surface. A perpendicular field can be applied using a coil with its axis perpendicular to the surface.
  3. Securely fix the sample to the test rig. Apply hot melt around the sample using a glue gun if necessary. The solidified glue can easily be removed after the experiments. NOTE: Steps 5.1 - 5.2 also apply here.

### 6. *In Situ* *BH* Measurements and Domain Imaging

1. **Connect up the *in situ* domain imaging system as shown in Figure 3.**
  1. Connect the sensor excitation coils to the power output of the *BH* analyzer. We used an in-house *BH* analyzer developed by the University of Manchester. A detailed description can be found in our previous publication 15.
  2. Connect the Hall sensor to the *H* input channel of the *BH* analyzer.
  3. Connect the sensor *B* coils to the *B* input channel of the *BH* analyzer.
  4. Connect the *H* and *B* outputs of the *BH* analyzer to two analogue input channels of the Midas DA BNC Breakout box (referred to as DA box hereafter) respectively ensuring that both inputs are set to the ground source (GS).
  5. Connect the Sync In of the high-speed camera to the Sync Out of the DA box.
  6. Connect the Trigger of the high-speed camera to the Trigger of the DA box.
2. Input the test parameters in the *BH* analyzer software. The cross sectional area of the sample should be entered in m^2^; in this case 6 x 10^-^^6^ m^2^.
3. **Set the data sync parameters as per the instruction of the DA software.**
  1. Set the sync out rate (2,000/s) to be the frame rate (500 frame/s) of the high-speed camera multiplied by the number of data points per frame (4 per frame).
  2. Set the pre-trigger length (in percentage) to be same as that of the camera.
4. Set the high-speed video camera ready for recording. That is, the camera will start waiting to be triggered.
5. Turn on the *BH* analyzer and apply a 1 Hz excitation sinusoidal current to measure the major loop; an image of the *BH* loop will be displayed.
6. Check that the measured *BH* loop is roughly as expected in terms of coercive field, remanence, magnetic saturation, etc. If it is not, the mechanical coupling between Part A and Part B should be inspected.
7. Trigger the camera either by sending a trigger signal from the *BH* analyzer or by clicking on the Trigger button on DA software interface.
8. Stop recording data and video after at least one *BH* loop cycle in the DA software.
9. Turn off the *BH* analyzer. CAUTION: Do not keep the electric current running through the sample for a long time especially if a direct current (DC) is used, as the current will heat up the sample and quickly dry the ferro-fluid.
10. Clean the sample and refresh for future analysis.

## Representative Results

Figure 4 shows two examples of high quality static domain patterns without any applied magnetic field for an industrial-grade pure iron and a low carbon steel respectively. One can see the DWs clearly in both materials and different types of patterns including *e.g. *packets of parallel (or 180°) and 90° DWs, in different grains. Owing to the good quality of polishing, there are no signs of random distortion of domain patterns due to subsurface damage caused by grinding; and the results show a strong link to the microstructure. For example, the 180° DW spacing (typically about 10 µm for pure iron and about 5 µm for the low carbon steel) increases with the grain size (approximately 200 µm for pure iron and 25 µm for the low carbon steel in mean equivalent circular diameter) and the domain patterns are dependent on the grain crystallographic orientation. It should be noted that the DW thickness as observed in Bitter patterns does not reflect the real Bloch DW thickness, which is estimated to be approximately 30 nm for pure iron^5^. The high uniformity of the pattern quality indicates that the application of the ferro-fluid was optimal.

Figure 5 illustrates a few examples of unsatisfactory results due to poor surface preparation, Figure 5**a** and **5b**, or if one fails to fix the sample securely during dynamic imaging or to level the sample. Note even a very small offset movement is obvious under the microscope. The video will go out of focus under the action of the applied field perpendicular to the sample surface as illustrated in Figure 5c; or the sample will oscillate laterally at the frequency of the applied field in the case of a parallel AC field being applied.

Figure 6 shows a series of domain images extracted from the DW movement process video at different points of the *in situ* measured *BH* loop. The video clearly shows a strong link between the DW movement processes and the position on the *BH* loop. For example, the transition of 180° DWs into 90° ones in region A occur near the 'knee' of the BH loop, i.e. between points 1 and 50 during magnetization; and the process reverses between points 225 and 250 during demagnetization, which indicates the domains rotating towards the applied field direction. It is interesting that the majority of 180° DWs in the bottom series of images do not move significantly. The reason for this is unclear. One possibility may be that the applied field direction, which happens to be approximately perpendicular to domain directions and therefore can neither cause the 180° DWs to move nor rotate the domains to align with the field direction. However, the segments marked in region B bulge leftwards and rightwards during magnetization and demagnetization respectively whilst in region C bulges only slightly leftwards. These phenomena seem to indicate there may be subsurface particles or inclusions disrupting the local domain directions to have component parallel with the applied field and hence move under its action. It is also indicative that the magnetization is not fully saturated. Further analysis of the domain direction and microstructural characterization of crystallographic orientation of the grain and of any subsurface particles are needed.

**Figure:**
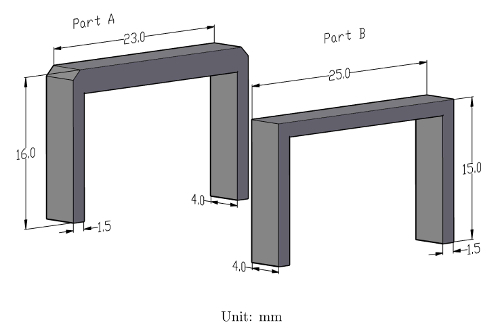

**Figure 1: Drawings of the sensor and specimen parts for *in situ* domain imaging (unit: mm).** Please click here to view a larger version of this figure.

**Figure:**
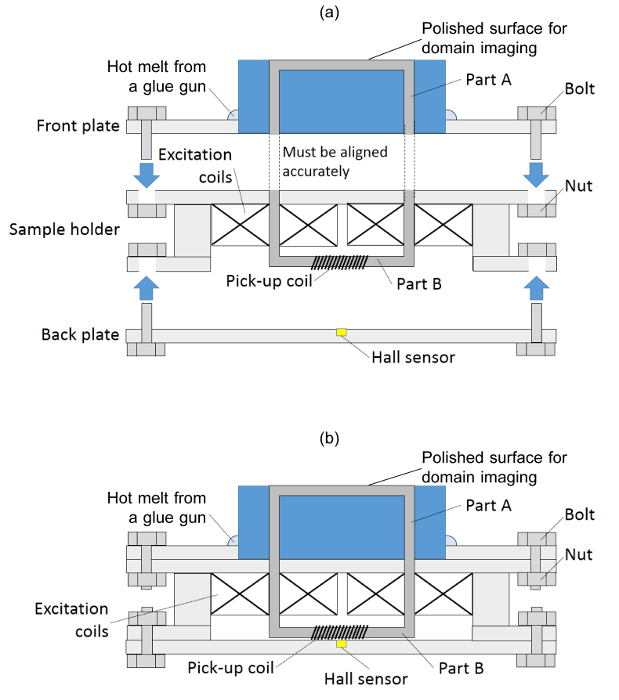

**Figure 2: Schematic assembly drawing of the *in situ* domain imaging rig 4. (a) **Separate parts before being assembled** (b) **finished assembly**. **Please click here to view a larger version of this figure.

**Figure:**
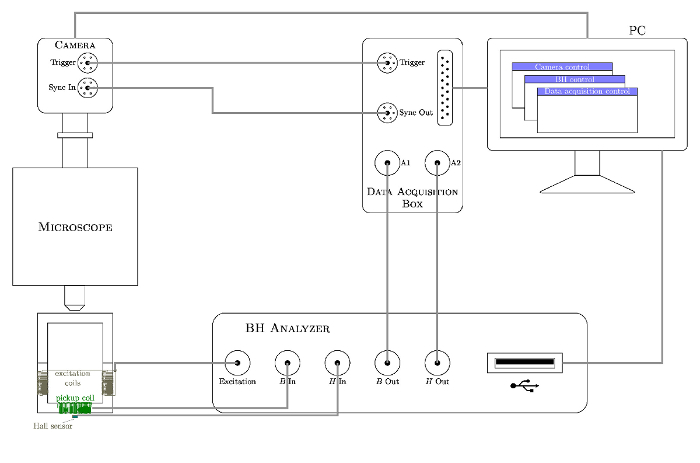

**Figure 3: Schematic of the components and connection of the *in situ* domain imaging system.** Please click here to view a larger version of this figure.

**Figure:**
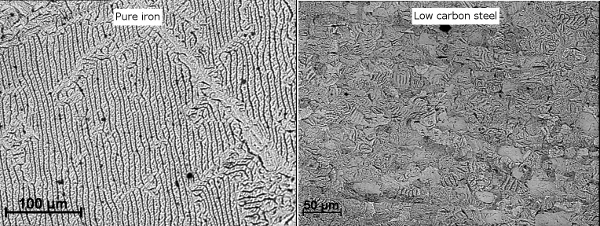

**Figure 4: Static domain patterns for pure iron and a 0.2 wt% carbon steel.** Please click here to view a larger version of this figure.

**Figure:**
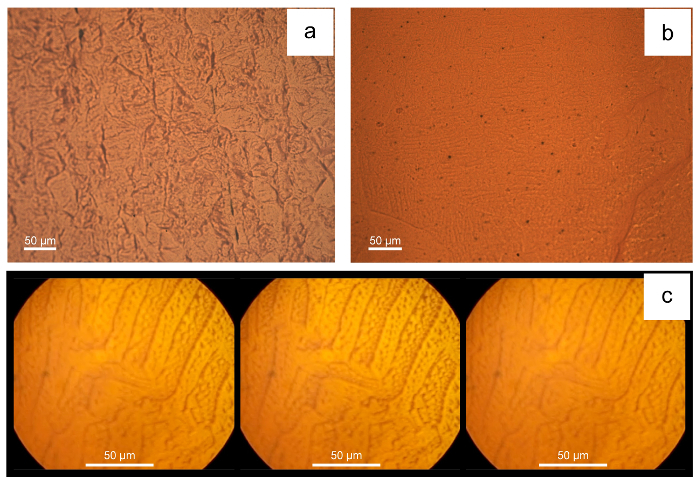

**Figure 5: Examples of unsatisfactory domain patterns resulting from failing to follow the protocols properly. (a) **disordered domain pattern (same low carbon steel sample as the one in Figure 3) lacking links to microstructure due to poor sample surface preparation;** (b**) obscure pattern with poor contrast due to poor application of the ferro-fluid on an as-cast extra-low carbon steel sample;** (c) **domain patterns going out of focus under the action of the perpendicular field of a pure iron sample Please click here to view a larger version of this figure.

**Figure:**
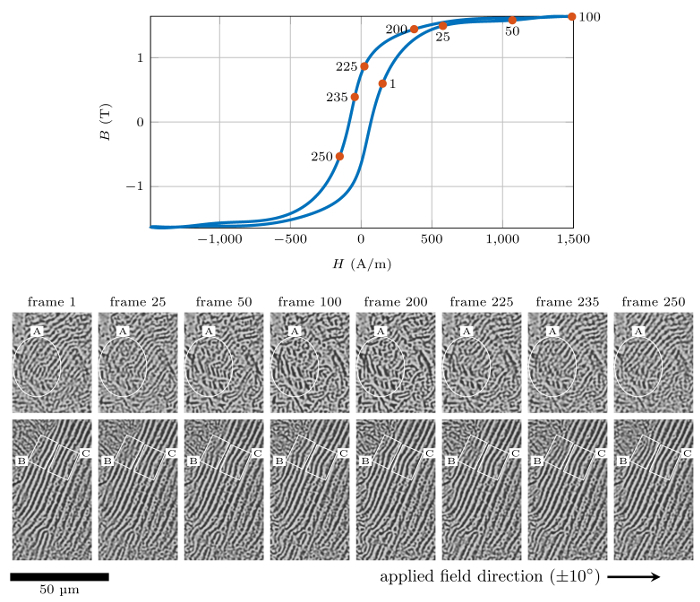

**Figure 6: A series of domain images extracted from the domain wall movement process video at frames corresponding to a series of points on the *in situ* measured *BH* loop with marked regions of interest showing domain rotation and likely interactions with microstructural features of an as-cast extra-low carbon steel sample.** Please click here to view a larger version of this figure.

## Discussion

The metallographic sample preparation is critical to the domain pattern quality by the Bitter method. The subsurface damage inherited from initial coarse grinding can obscure the real domain structure. These artificial effects usually result in poor contrast of DWs and many minor domain features associated with the strain due to the damage and sometimes a maze-like pattern. An amorphous surface layer may form due to serious surface damage, which will then give an unrepresentative domain structure. It is therefore important to take great care during grinding metallographic samples for domain imaging to minimize the subsurface damage in the first place. Additional procedures such as the etch-polishing cycles recommended in this paper or a long chemical mechanical polishing are often necessary to remove the remaining damaged surface layer. One needs to take extra care for sample preparation for the *in situ BH* measurement as excessive grinding or re-grinding will change the sample thickness; accurate thickness knowledge is required to determine the correct *B* values, as the flux density in Part A is inferred by the measurement of flux density in Part B. The *B* values outputted by the software are directly proportional to the cross-sectional area provided, so a 10% error in thickness will lead to roughly a 10% error in *B* values; the relationship is however non-linear, so a simple calibration after measurement is not possible. Over-ground samples can still be used for domain imaging but it should be noted that the measured *BH* loops will not be quantitatively representative of the real *BH* curve for the part of the sample being inspected. The *H *measurements should still be approximately representative of the real values whilst *B* values are smaller due to the reduced thickness and hence the cross-section area of the flat part. In the case of overgrinding, one can take the sample out of the mount to measure the thickness after all the domain imaging are completed and then scale the *in situ* measured B values (for the sensor) by a factor equal to the designed/final thickness to approximate the real *B* values (for the sample), only as a remedy measure.

The activity of the ferro-fluid is particularly important to dynamic domain imaging. If the degree of DW movements falls short of expectation one should check the ferro-fluid performance on a familiar sample using a DC applied field. If the issue remains, the ferro-fluid needs replacing. Fresh ferro-fluid is most active and it settles during storage. It is recommended to make a small amount of fresh ferro-fluid by dilution using original solvent for each experiment. The data on the activity of the ferro-fluid or the response time (to the change of the domain structure of the sample under examination) are not available whilst the latter is believed to be in the range of microseconds according to the supplier (Rene V, 2016). The frequency at which the magnetic field is applied for dynamic domain imaging in this investigation was 1 Hz, which is also the optimum frequency for major *BH* loop measurement. The performance of the ferro-fluid at higher magnetization frequency is yet to be assessed.

Whilst the Bitter method is convenient and sensitive its resolution is relatively low (about 1 µm) 11. This limits the application of the method for static domain patterns to steels that show DWs separate by >2 µm. However, it is still of value for dynamic domain imaging as the domain size increases under the action of the applied fields. The present test rig can only apply a field parallel with the sample surface for *in situ BH* measurements. To study the effect of crystallographic texture or the DW movement processes of grain-orientated steels one needs to consider the texture or grain orientation at the specimen sampling stage to ensure an appropriate sample orientation is chosen.

The significance of the *in situ BH* loop measurement is twofold. First, it enables quantitative interpretation of DW movement processes in terms of the applied field, and magnetic properties. Second, it helps establish a fundamental link between *BH* loop behaviors, magnetic properties and the microstructures of steels and ultimately helps interpreting EM sensor signals for microstructure evaluation. It is still challenging and of great significance to link the DW movement processes and/or domain structure to complex microstructures, particularly grain crystallographic orientations. In the future, electron back scattered diffraction (EBSD) analysis of the samples will be carried out and mapped to the static and dynamic domain patterns. The results will help interpret the different types of domain patterns observed in different grains and the different domain wall movement processes associated with the grain orientations with respect to the applied field directions.

When implemented correctly the *BH* loop produced by this method should be close to that produced using a closed magnetic circuit ring sample, as Parts A and B form a closed magnetic circuit. However, if both parts are not fitted perfectly together, an air gap will be introduced into the magnetic circuit and the results will be distorted. This distortion will present itself as *BH* loop shearing; a well-known effect characterized by an increase in maximum *H*, a decrease in magnetic remanence and the loop appearing more 'diagonal'. It is advisable to use the *BH* loop measurement system to acquire a *BH* loop using the Part A prior to mounting to compare to the loops acquired during the test, thus magnetic coupling can be assessed and repeatability optimized.

We chose the dimensions of the Part A and Part B considering the following factors and requirements. The reason for the differences of the Part A and Part B has been explained in Step 2.1. The mounting process described in Step 2 primarily dictates the horizontal length (25 mm, see Figure 1) of the samples used for these tests. A large polished surface area, determined by the horizontal length and the depth (4 mm, Figure 1) is beneficial to optical microscopy as well as sample preparation. The thickness of the sample should be the minimum required to produce a sufficiently rigid sample from the material under inspection; 1.5 mm in this case. The practicality and cost of machining should also be considered when choosing the thickness. The smaller the transverse cross section of the sample, the greater the flux density that can be generated by the excitation coils for a given current. Higher currents lead to more heat being generated and the ferro-fluid quickly drying out. A large number of turns of the excitation coils is desirable. The length of the two legs (15 mm, Figure 1) dictates the height of the rig. The latter must be smaller than the maximum spacing between the sample stage and the objective lens of the microscope. The maximum flux density and applied field are best decided by the user and are application specific. It is clear from observation when the *BH* loop is close to saturation (the *BH* loop exhibits a very small dB/dH), but this section of the curve stretches from very low applied fields to very high applied fields and could require values approaching 100 kA/m before the material could truly be said to be magnetically saturated. From our experience maximum applied field of 2 kA/m (for pure iron or soft steels e.g. all the steels studied in this paper) - 10 kA/m (for hard steels e.g. a martensitic steel) should magnetize the sample beyond the 'knee' of it major *BH *loop, during which most significant domain wall movements are expected to occur.

In summary, the present system for domain imaging with *in situ BH* measurement proved to be working for linking the DW movement processes directly to the *BH* loop of steels. This method is anticipated to become a useful tool for the fundamental study of microstructure-magnetic property relationships in steels, in conjunction with further microstructural characterization.

## Disclosures

The authors have nothing to disclose.
